# Mediterranean Native Leguminous Plants: A Reservoir of Endophytic Bacteria with Potential to Enhance Chickpea Growth under Stress Conditions

**DOI:** 10.3390/microorganisms7100392

**Published:** 2019-09-25

**Authors:** Clarisse Brígido, Esther Menéndez, Ana Paço, Bernard R. Glick, Anabela Belo, Maria R. Félix, Solange Oliveira, Mário Carvalho

**Affiliations:** 1 ICAAM—Instituto de Ciências Agrárias e Ambientais Mediterrânicas, Universidade de Évora, Pólo da Mitra, Ap. 94, 7002-554 Évora, Portugal; esthermenendez@uevora.pt (E.M.); apaco@uevora.pt (A.P.); afb@uevora.pt (A.B.); mrff@uevora.pt (M.R.F.); mjc@uevora.pt (M.C.); 2 IIFA—Instituto de Investigação e Formação Avançada, Universidade de Évora, Ap. 94, 7002-554 Évora, Portugal; 3 Department of Biology, University of Waterloo, Waterloo, ON N2L 3G1, Canada; glick@uwaterloo.ca

**Keywords:** diversity, functionality, manganese, salinity, rhizobia-legume symbiosis, plant-microbe interaction, symbiotic performance, plant growth promotion

## Abstract

Bacterial endophytes, a subset of a plant’s microbiota, can facilitate plant growth by a number of different mechanisms. The aims of this study were to assess the diversity and functionality of endophytic bacterial strains from internal root tissues of native legume species grown in two distinct sites in South of Portugal and to evaluate their ability to promote plant growth. Here, 122 endophytic bacterial isolates were obtained from 12 different native legume species. Most of these bacteria possess at least one of the plant growth-promoting features tested in vitro, with indole acetic acid production being the most common feature among the isolates followed by the production of siderophores and inorganic phosphate solubilization. The results of in planta experiments revealed that co-inoculation of chickpea plants with specific endophytic bacteria along with N_2_-fixing symbionts significantly improved the total biomass of chickpea plants, in particular when these plants were grown under saline conditions. Altogether, this study revealed that Mediterranean native legume species are a reservoir of plant growth-promoting bacteria, that are also tolerant to salinity and to toxic levels of Mn. Thus, these bacterial endophytes are well adapted to common constraints present in soils of this region which constitutes important factors to consider in the development of bacterial inoculants for stressful conditions in the Mediterranean region.

## 1. Introduction

The internal biota that exists within plant tissues includes a diverse range of microbes that have a significant influence on many attributes of the plant’s growth and development. Part of the microbial biota consists of endophytic bacteria that are able to colonize a plant’s internal tissues without causing any apparent harm to the host plant [1]. Moreover, these bacteria appear to play a key role in the normal as well as the stress functioning of host plants [2,3].

In these plant-microbe interactions, the plant host provides diverse protective niches for endophytic organisms while endophytic bacteria can benefit the host plants through the production of phytohormones, such as indoleacetic acid (IAA) and cytokinin, which are responsible for plant growth, development and biomass [4], increasing the uptake of nutrients like phosphorous or nitrogen [5], induce resistance or suppress phytophatogens [6,7], and increase resistance to abiotic stresses [8,9,10]. Additionally, these plant-microbe interactions contribute to the integrity, functioning, and sustainability of agroecosystems [11].

The Leguminosae (or Fabaceae) family has been identified as being a major component of the vast majority of habitats and sub-regions in the Mediterranean region as well as of the majority of the temperate regions of the world. The Mediterranean basin is thought to be the place where a number of legumes have originated [12]. In this regard, ~91 genera comprising 1956 species and 495 sub species of legume taxa are found in the Mediterranean region [13]. Hence, legumes are a key component of Mediterranean agro-ecosystems, both within the Mediterranean region as well as other regions of the world with a similar climate [14].

Although the Mediterranean region is characterized by a wide range of temperature and moisture supply, as well as by some soil mineral deficiencies and or toxicities, numerous legume species have adapted to grow under those conditions [15].

Considering that the association between legume plants and bacterial endophytes probably developed very early in plant evolution [1,16,17], and it is expected that this association occurs in all plant species [18], it can be speculated that the adaptation of native legumes to the Mediterranean conditions may be not only due to their genetics but also due to the establishment of different beneficial bacterial associations with soil microbes. From this point of view, native legumes are likely to be a reservoir of endophytic bacteria with a high potential for biotechnological and agronomic purposes. In this regard, we hypothesized that bacterial strains isolated from Mediterranean native legumes grown under appropriate conditions would also increase the growth of other legume crops, such as chickpea.

The overall aims of this work were (i) to isolate and identify cultivable endophytic bacteria from native legumes grown in Mediterranean soils, (ii) to characterize these strains in terms of common plant growth-promoting traits, and (iii) to investigate whether these endophytic bacteria are able to increase the symbiotic performance of rhizobia with their plant host and facilitate plant growth under a range of different environmental conditions. This study showed that Mediterranean native legume species harbor highly diverse endophytic communities in their root tissues and that most of these bacteria possess at least one plant growth-promoting feature, and are also tolerant to salinity and to toxic levels of Mn. Thus, they might contribute to the host’s adaptation to adverse environmental conditions in this region, and may be good candidates for the development of bacterial inoculants for stressful conditions.

## 2. Materials and Methods

### 2.1. Plant Material

Several native leguminous plant species common to the Mediterranean region were surveyed for the presence of plant growth-promoting endophytic bacteria. Legume plants were harvested from two different sites (±65 km distance among sites) in South of Portugal, namely in an acid Cambisol (granitic origin) under natural pasture at Herdade da Mitra, at the University of Évora (38°32′ N; 08°00′ W), known for its Mn toxicity to plants, including leguminous species [19,20], and from the ridge between two rice fields damaged by salinity located at Alcácer do Sal (38°25′ N; 08°36′ W). In addition to the differences in the soil types of these sites, they also possess distinct bioclimatic conditions: Herdade da Mitra is located in inferior subhumid and inferior mesomediterranean (ombrotype/thermotype) while Alcácer do Sal is under a superior dry and superior termomediterranean [21]. Healthy mature legume plants were randomly collected during the growing season (sampling was carried out in April 2014). Individual plants were put into plastic bags and taken to the laboratory where samples were stored at 4 °C until further processing, which was carried out within 48 h. The identification of leguminous plants species was done according to Flora Iberica [22,23].

### 2.2. Isolation of Culturable Endophytic Bacteria from Legume Roots

The roots of native leguminous plants were detached from the shoots and then thoroughly rinsed in tap water to remove any adhering soil particles. The root nodules that were visible were detached from the roots using a sterile clamp and were then discarded. Root surface sterilization and bacterial isolation were performed according to [24]. The efficiency of the root surface sterilization procedure was determined by the absence of any bacterial growth from 100 µL aliquot of the last rinsing water inoculated in Tryptic Soy agar (TSA) (Merck KGaA, Darmstadt, Germany) plates after 48 h at 28 °C incubation. Bacterial isolation was performed onto three different media as in [24]. For each legume species collected from same site, colonies with different morphologies were picked and sub-cultured 2−3 times until a pure culture was obtained and used for further analyses.

### 2.3. Screening for Plant Growth-Promoting Characteristics in vitro

The ability of the bacterial endophytic isolates to solubilize phosphate, produce indoleacetic acid (IAA), synthesize siderophores, possess cellulase activity and to inhibit the growth of *Fusarium oxysporum* f. sp. ciceri mycelium was assessed as previously described [24]. The isolates’ ability to produce siderophores and to solubilize phosphate was determined according to the formula ((colony + colored or clearance zone diameter)/colony diameter) described by [25], with an index >1 considered as positive for these abilities. The cellulase activity was determined by measurement of the clearing zone around the colonies. The antifungal activity was considered positive when inhibition of the mycelium development occurred compared to the mycelium growth in a negative control (i.e., plates inoculated only with the fungal agent). All of these experiments were performed in triplicate.

To evaluate the 1-aminocyclopropane-1-carboxylate (ACC) deaminase activity of the bacterial endophytes, two independent and sequential methods were performed adopting the strategies indicated previously in [26]. First, the isolates were grown overnight at 30 °C in Tryptic Soy Broth (TSB) (Merck KGaA, Darmstadt, Germany) and 50 µL of each growth culture was used to inoculate 2 mL of DF salt medium [27] without any nitrogen source and with ACC at a final concentration of 5 mM as the only nitrogen source. The cultures were grown at 30 °C on an orbital shaker for 48 h. Then, the cell culture growth under each condition was measured by reading the absorbance at 600 nm. The cell cultures with higher growth in DF salt supplemented with ACC than in DF salt without a nitrogen source were selected as putative ACC deaminase-possessing bacterial endophytes and used for subsequent ACC deaminase activity assay. The ACC deaminase activity of the endophytic bacterial isolates was determined as previously described [28], but using 4 mL of modified M9 minimal medium [29] to grow the bacterial cells for 48 h at 30 °C. ACC deaminase activity was determined by measuring the amount of α-ketobutyrate that formed following the cleavage of ACC by the enzyme ACC deaminase [30] in comparison with a standard curve of α-ketobutyrate. The protein concentration of the toluene-treated cells was determined by the method of Bradford [31]. The final ACC deaminase activity was expressed in μmol α-ketobutyrate/mg protein/h.

### 2.4. Tolerance to Salt, Aluminium, and Manganese

Endophytic bacteria tolerance to Mn and Al was evaluated based on their growth in 96-well microplates filled with 200 µL per well of modified M9 liquid medium supplemented with MnSO_4_ or AlK(SO_4_)_2_ at final concentrations 0.05, 0.1, 0.5, 1.0 and 2.5 mM. Similarly, tolerance to salt was evaluated using modified M9 liquid medium supplemented with NaCl at final concentrations of 85 mM (0.5%), 425 mM (2.5%), 850 mM (5.0%), 1.275 M (7.5%) and 1.7 M (10%). For each bacterial isolate, a 20 µL aliquot (OD_600 nm_ = 0.05) was inoculated into a well of a 96 well microplate. Non-inoculated medium served as a blank. The 96 well microplates were incubated with shaking at 30 °C. Following 2 days incubation, the absorbances of each of the wells of the microplates were read at 600 nm using a microplate reader (Multiskan spectrum, Thermo Scientific, Waltham, MA, USA). The maximum concentration of salt, Al or Mn that was tolerated for each bacterial endophyte was considered to be the concentration that showed at least 75% of their growth under control conditions (i.e., modified M9 liquid medium without the addition of Mn, Al or NaCl).

### 2.5. Gnotobiotic Root Elongation Assay

For the gnotobiotic root elongation assay, a non-legume plant generally used to conduct gnotobiotic systems for studying plant growth promotion activities by bacteria, as the case of canola, was chosen. The ability of endophytic bacteria to promote the elongation of canola roots was carried out and monitored as described by [30]. Briefly, the bacterial cell pellet obtained from an overnight growth in liquid M9 minimal medium was suspended in 0.5 mL sterile 0.03 M MgSO_4._ Then, the absorbance of each sample was measured at 600 nm and the bacterial suspension was adjusted to an OD_600 nm_ of 0.15 with sterile 0.03 M MgSO_4_.

Surface sterilized canola seeds (*Brassica campestris*) were disinfected immediately before use by soaking in (i) 70% ethanol for 1 min then (ii) 1% sodium hypochlorite for 10 min, followed by (iii) several washes with sterile distilled water. A bacterial aliquot was added to each group of seeds and incubated at room temperature for 1 h. As a negative control, a group of seeds inoculated only with sterile 0.03 M MgSO_4_ was used. Following the 1 h incubation period, six bacterized seeds were placed in each growth pouch using sterilized forceps, with 10 growth pouches being used for each treatment. Subsequently, the growth pouches were incubated in a plant growth chamber for 5 days before the primary root lengths were measured and recorded.

### 2.6. 16S rRNA Gene Sequencing and Phylogenetic Analysis

Total DNA extraction of bacterial endophytes was performed as previously described [24]. The 16S rRNA gene of each isolate was amplified using one set of the following primer pairs: Y1 [32] and Y3 [33] or b341 [34] and 1193R [35]. The PCR program and conditions for reactions containing the Y1 and Y3 primers set were as described earlier [24]. The PCR reactions, containing the b341 and 1193R primers set, were performed in a final volume of 50 µL as follows: 1X DreamTaq Buffer, 0.2 mM of each dNTP, 25 pmol of each primer, 1−10 ng bacterial DNA, 1.25 U DreamTaq DNA polymerase (Thermo Fisher Scientific Inc., Waltham, Massachusetts, USA). The amplification program consisted of: 5 min at 95 °C as an initial denaturation, followed by 35 cycles of 1 min at 95 °C, 90 s at 52 °C and 100 s at 72 °C; with a final extension of 7 min at 72 °C. The resulting amplified DNAs were purified using DNA Clean & Concentrator™-5 Kit (Zymo Research, Irvine, California, USA) according to the manufacturer’s instructions, and were subsequently sequenced by Macrogen, Inc. (Seoul, Korea) using the universal primer 1100R [36], or by StabVida Lda (Lisboa, Portugal) using the specific primers b341 and 1193R.

The sequences obtained were compared with those from the GenBank and EzBioCloud public databases [37]. Sequences of partial 16S rRNA gene (~650−750 pb) were aligned using the ClustalW [38] and distances were calculated according to Kimura’s two-parameter model [39]. The phylogenetic analysis was constructed using MEGA7 [40] with the Neighbour-Joining model [41] with 1000 bootstrap replications. The circular phylogenetic tree was drawn from the newick archive generated by MEGA 7 using the online Interactive Tree of Life (iTOL) v4 tool [42]. The partial 16S rDNA nucleotide gene sequences that were obtained in this study were deposited in the NCBI GenBank database under the accession numbers MK007348 to MK007469.

### 2.7. Plant Growth Promotion Assays under Control and Stress Conditions

In order to evaluate the effect of bacterial endophytes on the symbiotic performance of rhizobia with their plant host, plant growth assays using chickpea plants grown under controlled conditions including salinity, and manganese were performed.

Salinity and Mn toxicity were chosen as stressing conditions due to their impact in reducing crop yields worldwide. Over 40% of the world’s arable land suffers from soil acidity and, therefore, Al and Mn toxicities [43]. Similarly, salinity affects around 40% of the world’s land surface, with the arid and semiarid regions particularly prone to salinization [44]. Moreover, both symbiotic partners of the rhizobium-legume symbiosis present different degrees of sensitivity to these stresses. Legumes are generally more sensitive to salinity than their microsymbionts [44], while rhizobia are more sensitive to heavy metals than their legume hosts, with some legumes being used in phytoremediation due to their high tolerance to metals [45,46].

Chickpea (*Cicer arietinum* L.) was chosen for these experiments due to its importance in terms of a food legume crop and source of protein worldwide, whose production is limited by several biotic and abiotic stresses [44]. Moreover, two compatible mesorhizobia strains, namely *Mesorhizobium ciceri* LMS-1 [47] and *M. mediterraneum* UPM-Ca36^T^ [48] strains, were selected in order to evaluate whether the co-inoculation effect with the endophytic bacteria is dependent on the *Mesorhizobium* strain used. Based on plant growth-promoting characteristics, three *pseudomonads* strains, *Pseudomonas* sp. D4, L13 and Q1, from this work, were chosen to perform these experiments. Additionally, the endophytic bacterial strain *Kosakonia* sp. MH5, which was previously isolated from chickpea roots [24] was chosen to evaluate whether an endophytic bacterial host origin would affect the final outcome of co-inoculation in chickpea plants. The treatments were as follows: (i) uninoculated chickpea plants either mesorhizobia or endophytic bacteria (ii) chickpea seeds inoculated with *M. ciceri* strain LMS-1 or *M. mediterraneum* UPM-Ca36^T^ alone, (iii) *Mesorhizobium* strain combined with each endophytic bacterial isolate, (iv) three plant growth conditions: control, salinity and manganese.

The *Mesorhizobium* inoculum was prepared as previously described by [49], with the exception that cells were washed twice with sterile saline solution (0.85% NaCl), and the optical density was recorded at 600 nm. A similar procedure was conducted for the endophytic bacterial inoculum, where the bacterial cells were cultured overnight in TSB medium at 28 °C, followed by two washes with sterile saline solution and then resuspended in the same solution.

Chickpea seeds were surface sterilized and germinated as described [48]. One seed was planted per plastic pot (±300 mL) previously filled with sterilized vermiculite. One mL of the bacterial suspension (final OD_600nm_ of 0.8) was used to inoculate each germinated seedling. For co-inoculation, cell suspensions of two bacterial isolates were mixed in a 1:1 ratio, i.e., containing a final OD_600nm_ of 0.8 of each bacterium.

A nitrogen-free nutrient solution [50] was used to simulate control and stress conditions. The regular nutrient solution was used for control conditions. The nutrient solution supplemented with 0.15% NaCl, as previously described [49], was used for salinity conditions whereas the nutrient solution with a final 30 ppm of Mn was used for manganese conditions. Uninoculated plants were watered with the respective nutrient solution for each growth condition and supplemented with nitrogen (0.1% KNO_3_) were used as positive controls. The plant growth assays were conducted in a growth chamber with the temperature, light and relative humidity parameters (a 16-h-light and 8-h-dark cycle and 24 °C-day and 18 °C night temperature at a relative humidity of 65%) set as in [47]. Each treatment consisted of five plant replicates. Plants were harvested after six weeks when the total biomass (shoot + root dry weights) and nodulation parameters were determined.

### 2.8. Statistical Analysis

Statistical analyses were performed using SPSS 25.0 software (SPSS Inc; IBM New York, USA). The data obtained from gnotobiotic root elongation and chickpea plant-growth assays were characterized by analysis of variance (ANOVA). The post hoc Duncan test was used for multiple comparisons of root length means and the independent samples t-test (*p* < 0.05) for comparison of means of plant and nodulation parameters.

To investigate whether the isolate’s ability to synthesize IAA, produce siderophores, solubilize phosphate or produce cellulase activity were related to bacterial genera, origin site or legume species, the IAA production, cellulase activity, and siderophore and phosphate index values were divided into classes. The levels of IAA production were divided into five classes [no IAA production; low level of IAA production (<5 µg mL^−1^); medium level of IAA production (between 5 and 15 µg mL^−1^); high level of IAA production (between 15 and 50 µg mL^−1^); and very high level of IAA production (>50 µg mL^−1^)]. Four different levels of siderophore production were defined [no production (=1), low level of production (>1 and <1.5), medium level of production (≥1.5 and <2.5), and high level of production (≥2.5)]. Similarly, the isolate’s ability to solubilize phosphate was classified into three categories according to the index value [no solubilization; low level of solubilization (>1 and ≤1.5) and high level of solubilization (>1.5) ]. According to the Zones of clearance around the colonies, four different levels of cellulase activity were considered [no activity; low activity (>0 and ≤0.5 cm); high activity (>0.5 and ≤1 cm) and very high activity (>1 cm)]. Relationships between the category variables were determined using the Chi-Square test of association. Fisher’s exact test was used when category variables include small sample sizes (*n* < 5).

Principal components analyses (PCA) were performed using the PRIMER version 6 with PERMANOVA+ [51,52] (PRIMER-E Ltd, Plymouth, UK) to evidence that the effects of specific endophytic bacterium on plant and nodulation parameters were *Mesorhizobium* strain- or plant growth condition-dependent. The data means of plant biomass, nodule dry weight and number of nodules were log transformed and normalized prior to conduct PCA using Mesorhizobia strains and plant growth conditions as factors.

## 3. Results

### 3.1. Native Legumes Surveyed

A total of twelve native legume species were surveyed in this study, covering the genera *Lupinus, Ornithopus, Scorpiurus, Trifolium, Medicago* and *Vicia* (Table 1). From these, four plant species, namely *Lupinus luteus, Ornithopus compressus, Medicago polymorpha* and *Vicia sativa* were commonly found in both sites. Legume species belonging to the genera *Trifolium* and *Scorpiurus* were exclusively found in the Herdade da Mitra site while the species *Ornithopus sativus* and *Lupinus angustifolius* were only found in the Alcácer do Sal site.

### 3.2. Bacterial Endophytes Diversity and Distribution Among Legume Host Species and Sites

A total of 122 endophytic bacteria were isolated from the roots of the native legume species collected from the two different locations in the South of Portugal (Table 1).

**Figure 1:**
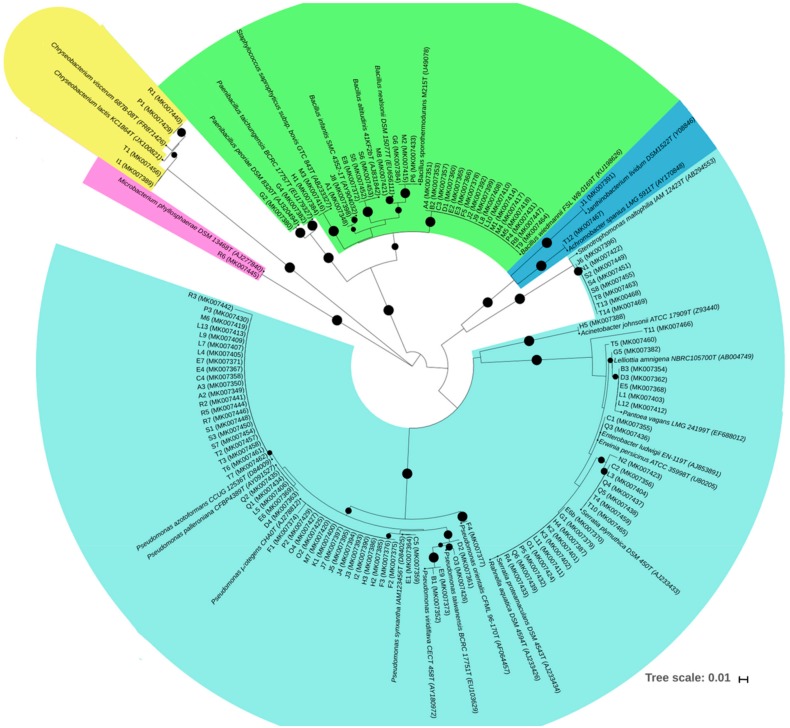
Neighbor-joining phylogenetic tree based on partial 16S rRNA gene sequences (approx. 700 pb) of 122 bacterial endophytes and their related type strains (accession numbers included in brackets). The evolutionary distances were computed using the Kimura 2-parameter method. Black nodes mean bootstrap percentages higher than 50%, which are based on 1000 replicates. Light blue- Gammaproteobacteria class; Dark blue- Betaproteobacteria class; Green- Firmicutes phylum; Yellow- Bacteroidetes phylum; Pink- Actinobacteria phylum.

Based on the 16S rRNA nucleotide sequences obtained from those bacterial endophytes, four phyla, namely Proteobacteria (71.3%), Firmicutes (24.6%), Bacteroidetes (3.3%) and Actinobacteria (0.8%), were identified (Figure 1). Within the phylum Proteobacteria, bacterial endophytes were assigned to six families with the Pseudomonadaceae and Enterobacteriaceae families being the most represented with 56.3% and 31% of the total number of bacterial isolates, respectively. Although they were in relatively low abundance (<2.2%), isolates assigned to *Janthinobacterium, Acinetobacter, Enterobacter, Erwinia, Lelliottia* and *Achromobacter* genera were also identified. *Bacillus* was the most predominant genus within the phylum Firmicutes while only one genus were recorded within the phyla Actinobacteria and Bacteroidetes, namely *Chryseobacterium* and *Microbacterium*, respectively.

**Figure 2:**
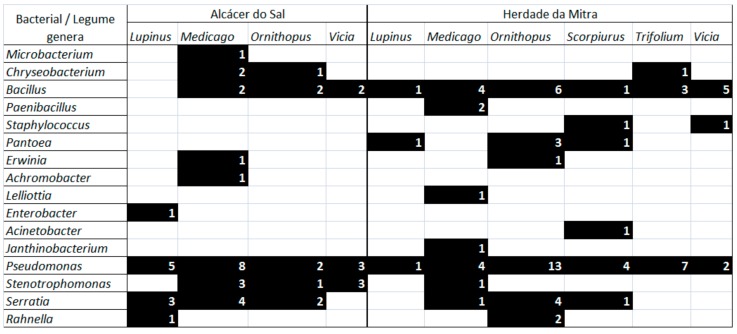
Distribution of the bacterial genera according to legume genera and origin region. Number of isolates assigned to each bacterial genus are indicated. Black shadow indicates presence of bacterial genus.

Bacterial endophytes belonging to the genus *Pseudomonas* were found in all legume species, independent of their origin site, with the exception of *Ornithopus compressus* which was only found in plants from the Herdade da Mitra site (Figure 2; Table S1). The second most widespread genus was *Bacillus*, which was found in eight of the twelve plant species analyzed and in all six plant genera. On the other hand, the genera *Microbacterium, Janthinobacterium, Lelliottia, Achromobacter,* and *Paenibacillus* were solely obtained from *M. polymorpha* roots and the genus *Acinetobacter* was exclusively found in *S. sulcatus* roots.

*M. polymorpha* and *O. compressus* plant species presented the greatest diversity of endophytic bacterial genera while *O. pinnatus* and all *Trifolium* species showed the least diversity. While there were no significant differences between the total number of bacterial genera obtained at the two collection sites, the presence of the various genera identified was not equally detected in the plants harvested in Herdade da Mitra site and those that were collected from Alcácer do Sal (Figure 2, Table S1). In fact, the diversity of bacterial endophytes was significantly different among the two sites (χ^2^ = 28.159; d.f. = 14; *p* < 0.01; Fisher’ Test *p* < 0.01). For instance, bacterial endophytes assigned to the genera *Staphylococcus, Paenibacillus, Janthinobacterium, Lelliottia, Acinetobacter* and *Pantoea* were only found in legume roots collected in the Herdade da Mitra site whereas *Enterobacter, Microbacterium* and *Achromobacter* were only obtained from legume plants grown in the Alcácer do Sal site.

### 3.3. Potential of Bacterial Endophytes for Plant Growth Promotion and Cellulase Production

The bacterial endophytes that were isolated from legume roots were evaluated for their cellulase activity and plant growth promotion potential, namely the synthesis of indoleacetic acid (IAA), siderophore production, phosphate solubilization, antifungal and ACC deaminase activity (Table S1). Fifty two out of 114 (45.6%) bacterial strains showed positive results for cellulase activity. Similarly, more than half of the strains (55.5%) synthesize IAA-like molecules at different levels ranging from 0.1 to 97.1 µg mL^−1^ (Table 2, Table S1).

**Figure 3:**
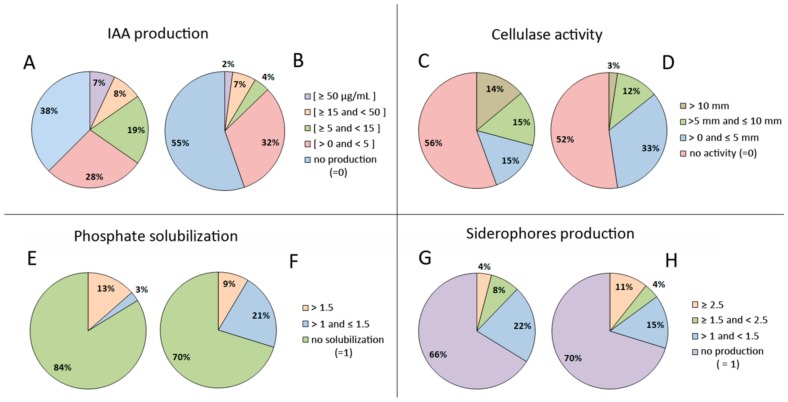
Percentage of isolates displaying cellulase activity and different plant growth-promoting traits: inorganic phosphate solubilization, IAA and siderophore production. (**A**,**C**,**E**,**G**) correspond to isolates obtained from legume grown in Herdade da Mitra and (**B**,**D**,**F**,**H**) from Alcácer do Sal. The percentage of isolates are distributed according to the level of IAA production, cellulase activity classes and index of siderophore production and phosphate solubilization.

This feature was the most common plant growth-promoting trait among the isolates. On the other hand, only 32% of the isolates tested have the ability to produce siderophores, but almost half of these were good siderophores producers, with a siderophore production index ≥1.5 (Figure 3).

According to the levels of cellulase activity, IAA or siderophore production, associations between these levels and the isolate’s genera were found (χ^2^ = 98.457; d.f. = 42; *p* < 0.001; for cellulase activity; χ^2^ = 180.854; d.f. = 56; *p* < 0.001 for IAA production; χ^2^ = 61.251; d.f. = 30; *p* < 0.01 for siderophore production). For instance, most of the isolates assigned to *Bacillus* and *Pseudomonas* were no or low IAA producers while *Erwinia, Chryseobacterium, Pantoea* and *Rahnella* isolates produced medium to high amounts of IAA-like molecules. Only isolates assigned to *Paenibacillus*, *Pseudomonas*, *Staphylococcus* and *Stenotrophomonas* were good siderophore producers and some of the isolates from *Bacillus, Chryseobacterium, Microbacterium, Paenibacillus, Pseudomonas, Rahnella* and *Stenotrophomonas* genera presented high cellulase activity. Although no statistical significance was found between the mean of IAA or siderophores produced and the legume species, the ability of strains to produce high amounts of IAA or siderophores was influenced by the legume host species (Table S1). The strains producing the higher amounts of IAA were isolated from *L. luteus, M. polimorpha* and *O. compressus* whereas most of the bacterial endophytes isolated from *V. sativa, L. angustifolius, O. sativus* and *S. muricatus* didn’t synthesize IAA. In contrast, none of the bacterial strains isolated from *L. luteus* and *S. muricatus* were able to produce siderophores while the good siderophore producers were isolated from *M. polymorpha, O. sativus, L. angustifolius, S. sulcatus, S. muricatus* and *V. sativa*. On the other hand, the mean amount of IAA produced by endophytic bacteria obtained from legumes grown in the Herdade da Mitra site was significantly different from the amount obtained from strains isolated from the Alcácer do Sal site (*p* < 0.05). In fact, the mean of IAA production by the strains isolated from *M. polymorpha* or *V. sativa* harvested in Herdade da Mitra was 4- or 9-fold higher than the mean IAA production produced by the strains obtained from the same host species in Alcácer do Sal.

The ability of endophytic bacteria to solubilize inorganic phosphorous was not a common plant growth-promoting feature among these isolates (Table 2; Table S1); only 22% of isolates were able to solubilize inorganic phosphorous. Nevertheless, more than half of them were very good phosphorous solubilizers. Interestingly, there are a higher proportion of phosphate solubilizers from legumes harvested in the Alcácer do Sal region, but the phosphate solubilizers obtained from the Herdade da Mitra plants are mostly better solubilizers (index > 1.5) (Figure 3).

The ACC deaminase and antifungal activities were detected in 8 and 13 bacterial endophytes, respectively (Table S1). Moreover, some of these isolates exhibited both good ACC deaminase and antifungal activities. For example, the *Pseudomonas* sp. Q1 strain simultaneously showed high ACC deaminase activity (8.75 µmol.mg^−1^·h^−1^) and inhibited the mycelium growth of *Fusarium oxysporum* f. sp. ciceri agent to a great extent, indicating a high antifungal activity against this pathogenic fungus. Curiously, all bacterial endophytes with antagonistic activities against *Fusarium oxysporum* f. sp. were isolated from *O. compressus, M. polymorpha*, and *L. angustifolius*.

Nearly 83% of the endophytic bacteria isolated in this study possess one or more of the plant growth-promoting traits tested. A significant relationship between the number of plant growth-promoting traits and an isolate’s genera was observed (χ^2^ = 68.663; d.f. = 45; *p* < 0.05; Fisher’ Test *p* < 0.05). Thus, most of the isolates assigned to *Serratia, Pseudomonas* and *Bacillus* displayed a higher number of plant growth-promoting traits. However, no significant association between the number of plant growth-promoting traits and the legume species or site of origin was found.

### 3.4. Salt and Metals Tolerance and Promotion of Canola Root Length

Based on the level of cellulase activity and the results of the plant growth-promoting traits, 15 bacterial endophytes were selected for further characterization, namely tolerance to salt, manganese, and aluminium as well as promotion of root length of canola seedlings (Table 3). With the exception of *Pseudomonas* sp. Q2 and *Serratia* sp. G1, all of the remaining bacterial endophytes that were selected were tolerant to high concentrations of salt (≥850 mM NaCl), independently of isolates’ origin site. Similarly, most of the bacterial endophytes were also tolerant to high concentrations (2.5 mM) of manganese (69%) or aluminium (53%). However, only a few strains had a high tolerance to both manganese and aluminium, suggesting that the tolerance to these metals occurs by different mechanisms.

**Figure 4:**
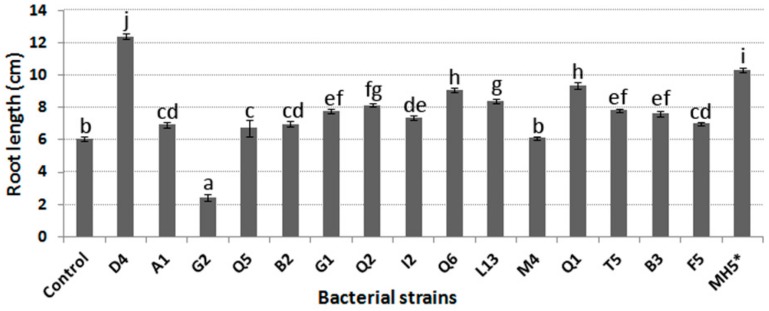
Results obtained from the gnotobiotic root elongation assay. Data correspond to the mean and standard error values of 25 plants replicates. Control corresponds to uninoculated seeds. Different letters (a-j) correspond to statistically significant differences (*p* < 0.05). * The endophytic bacterium strain MH5 was obtained from chickpea roots from a previous study [24].

All of the selected endophytic bacteria, with the exception of *Paenibacillus* sp. G2 and *Bacillus* sp. M4, significantly increased the root length of canola seedlings when compared to the root length of uninoculated seeds (Figure 4).

For instance, the length of canola roots inoculated with specific endophytic bacteria such as *Pseudomonas* sp. D4 or Q1 strains was 120% or 54.5% higher, respectively, than the root length of uninoculated seeds. Not surprisingly, these two endophytes possess ACC deaminase activity, which is known to promote canola root length elongation. Notwithstanding the fact that some of the selected endophytes show plant growth-promoting characteristics, some of them may be inhibitory to plant growth when they are inoculated alone, as was the case with *Paenibacillus* sp. G2.

### 3.5. Effect of Non-Rhizobial Endophytes on Symbiotic Performance of Mesorhizobium—Chickpea under Control and Stress Conditions

Based on the plant growth-promoting characteristics detected herein, four selected isolates (*Pseudomonas* sp. L13, *Pseudomonas* sp. Q1, *Pseudomonas* sp. D4 and *Kosakonia* sp. MH5) were evaluated for their ability to improve chickpea mesorhizobia symbiotic performance under control and stress conditions (Table 4). *Pseudomonas* sp. Q1 and *Pseudomonas* sp. D4 were chosen for possessing ACC deaminase activity in free-living conditions whereas *Pseudomonas* sp. L13 and *Kosakonia* sp. MH5 do not. *Kosakonia* sp. MH5 was the one able to produce detectable amounts of IAA and L13 and Q1 strains displayed antifungal activity against *Fusarium* sp. All strains could significantly increase canola root length and produce siderophores. Despite the fact that *Pseudomonas* sp. L13 and D4 were obtained from legumes grown in Mn toxic soil while *Pseudomonas* sp. Q1 and *Kosakonia* sp. MH5 were from legumes grown in saline soils, all of them present tolerance to both NaCl and Mn. Prior to conducting the plant growth experiments, the isolates were inoculated in chickpea seedlings and re-isolated from surface sterilized chickpea roots 7-days after inoculation to ensure their ability to colonize the interior of chickpea roots.

As expected, both stress conditions imposed were responsible for a significant reduction of the total biomass of uninoculated plants compared to those grown under control conditions. However, this reduction was more pronounced in the case of salinity than in the presence of manganese, indicating a higher tolerance to the presence of manganese toxicity than to salt stress. On the other hand, the *Mesorhizobium*-chickpea symbiosis was more severely affected by manganese than by salinity.

Under control conditions, there were no significant differences between the total biomass of co-inoculated plants and the total biomass of plants single inoculated with *Mesorhizobium mediterraneum* UPM-Ca36^T^. Although there was a trend towards an increase in the total biomass of plants co-inoculated with any of the consortia containing *M. ciceri* LMS-1 and each non-rhizobial endophyte, only the total biomass of plants inoculated with the LMS-1 and *Pseudomonas* sp. L13 strains was statistically higher than the total biomass of plants inoculated only with LMS-1. Regarding the nodulation abilities, the number of nodules formed by either LMS-1 or UPM-Ca36^T^ when co-inoculated with *Pseudomonas* sp. D4 and by LMS-1 with (*Pseudomonas* sp. L13 decreased significantly (*p* < 0.05) when compared to the number of nodules formed by these strains when inoculated alone, however without significantly affecting the total nodule dry weight. In fact, those nodules formed by the rhizobial strains in the presence of other endophytic bacteria were heavier (average weight per nodule) than the ones formed in single inoculated plants.

In terms of plant growth promotion under saline conditions, positive effects on total biomass of chickpea plants inoculated with distinct consortia containing either LMS-1 or UPM-Ca36^T^ symbionts were observed (Table 4). The endophytes *Pseudomonas* Q1 or L13 strains combined with any of the rhizobial strains resulted in significant promotion of the chickpea biomass compared to plants single inoculated with *Mesorhizobium*. The total biomass of plants co-inoculated with L13 and any of the *Mesorhizobium* symbionts was increased 25% compared to singly inoculated plants whereas an augmentation of 10 or 27% was observed in plants co-inoculated with Q1 and LMS-1 or UPM-Ca36^T^, respectively. In contrast, significant increase of chickpea biomass by the consortium containing the MH5 or D4 strains was *Mesorhizobium*-dependent (Figure S1). For instance, inoculation of chickpea plants with the endophytic MH5 strain only significantly improved the chickpea biomass when co-inoculated with the LMS-1 symbiont while the D4 strain significantly improved the chickpea biomass when co-inoculated with the UPM-Ca36^T^ strain. The total biomass increased >35% compared to singly inoculated plants with the respective *Mesorhizobium* strain. In contrast to what was observed under control conditions, a trend towards an increase in the number of nodules formed by either *Mesorhizobium* symbionts was observed under saline conditions. The co-inoculation of LMS-1 with MH5 or L13 strains significantly improved the nodulation of chickpea more than 1.5-fold. Similarly, the co-inoculation of UPM-Ca36^T^ with all endophytic strains with the exception of the MH5 strain significantly increased the total nodule dry weight by more than 32%. Interestingly, the promotion of plant growth under salinity occurred either with bacterial endophytes originating from saline or toxic manganese soils, suggesting that endophyte-plant synergism was not dependent on the origin of the bacterial endophytes isolates but probably on the complementarily of plant growth-promoting mechanisms along with the N_2_-fixing symbionts.

Curiously, none of the endophytic bacteria in combination with the LMS-1 symbiont significantly improved chickpea biomass or the nodulation abilities under manganese conditions (Table 4). The inoculation of the UPM-Ca36^T^ symbiont with the endophytic Q1 strain significantly increased the total biomass by more than 40% compared to the total biomass of single inoculated plants. On the other hand, the presence of *Pseudomonas* sp. Q4 with UPM-Ca36^T^ resulted in the opposite results, i.e., a significant reduction of the total biomass, most probably due to the significant decrease in the number of nodules formed in those plants leading to a significant reduction of total nodule dry weight. The increase of plant biomass observed in plants co-inoculated with the Q1 or L13 endophytes with UPM-Ca36^T^ may be a result of the significant augmentation of the total nodule dry weight in those plants compared to those inoculated only with UPM-Ca36^T^. Similar to saline conditions, the bacterial endophyte origin was not related to the improved chickpea biomass, since the beneficial effects observed were from an endophyte that originated from saline soil whereas the reduction of chickpea growth was noticed when plants were co-inoculated with UPM-Ca36^T^ and *Pseudomonas* sp. Q4, an endophytic bacterium that originated from Mn toxic soils.

## 4. Discussion

In the present study, the diversity and functioning of bacteria living within the roots of native legume species growing in two sites (with a geographical distance of ±65 km and for possessing different soil types and distinct bioclimatic conditions) in the South of Portugal were investigated. Moreover, the ability of these isolates to improve chickpea mesorhizobia symbiotic performance under control and stress conditions was also evaluated.

A collection of 122 bacterial endophytes from 12 different native legume species was obtained in this study. Although the total diversity of the endophytic bacterial communities of these plant legumes were not specifically investigated herein due to culture-dependent method limitations [53], the results nevertheless covered four phyla, Proteobacteria, Firmicutes, Actinobacteria and Bacteroidetes, which is consistent with the bacterial diversity usually detected in the in legume roots or root nodules [54,55,56]. Although a portion of the slow-growing and non-culturable endophytic bacteria may have been excluded in this work due to culture-based method used, our data clearly indicate that Mediterranean native legumes harbor diverse endophytic bacterial communities.

The extensive prevalence of phylum *Proteobacteria* in root tissues is in accordance with the results of other studies [55,56,57,58]. While *Pseudomonas* was the most abundant genus, the genera *Microbacterium*, *Janthinobacterium*, *Lelliottia*, *Acinetobacter* and *Achromobacter* were the rarest. Moreover, *Pseudomonas* was the only genus common to all legume species which agrees with the fact that *Pseudomonas* is one of the most dominant genera in plant microbiomes [59]. In addition, a recent study of native legume species in Portugal also revealed the predominance of this genus in the legume root nodules [60]. However, the bacterial diversity found in legume root nodules is considerably different from the diversity found in the legume roots studied herein. The difference in the culture media used in these two studies, which is known to influence the apparent composition and diversity of the cultured bacterial community [61], may account for the differences. Another explanation for the difference between results is that the endophytic bacterial communities that exist in root nodules may be different from those that colonize the interior of legume roots. In fact, a clear differentiation between non-rhizobial bacterial community composition in legume roots and nodules were previously observed in *Lotus japonicus* [62], soybean and alfalfa [56]. For instance, several genera, like *Azospirillum*, *Flavobacterium*, *Lysobacter*, *Variovorax*, *Agrobacterium*, *Microvirga,* and *Phyllobacterium* [60,63,64,65,66,67] that are typically found in root nodules from distinct legumes species were not detected in f the roots of the legume species studied here.

It has been pointed out that distinct factors, like plant host genotype, soil type, environmental soil conditions, and cultivation history, shape the bacterial communities associated with different plants [62,68,69,70,71,72]. While our data do not allow for the elucidation of the influence of legume species on endophytic bacterial diversity due to the low number of isolates obtained in some of the legume species, it was nevertheless possible to observe that the diversity of endophytic bacteria found in the two sites was different. This may be due to differences in the soil type of these sites or to plant genotypes or to a combination of both. Indeed, it was previously observed that the soil types shape the bacterial community composition in the rhizosphere [73], the most important source of endophytes [74]. Similarly, plant genotype determines rhizospheric community composition [70,73,75], and consequently exerts direct pressure over the bacteria that colonize the root surface and subsequently within plant tissues as endophytes. However, the buffering effect on the rhizosphere population varies among plant species [70], and can be overcome in some cases by soil type or geochemistry [69,76]. Overall, the results presented here may be explained by factors that influence soil microbiome by changing the diversity and composition of the bacterial communities available to colonize the interior of plant root tissues.

It is believed that plants actively select their microbiota [70,77,78] throughout their life in order to recruit microbes that may help them to cope with changing environmental conditions and facilitate adaptation to stress. Here, it was hypothesized that the adaptation of native leguminous species to Mediterranean climatic conditions could be facilitated by their association with endophytic bacteria. Therefore, these legume species were predicted to be a reservoir of plant growth-promoting bacteria with potential for ecological and agricultural purposes.

Indeed, the data presented here show that 101 out of 122 bacterial endophytes possess at least one of the plant growth-promoting traits tested, the production of indoleacetic acid (IAA) being the most widespread trait among the strains. Furthermore, the best IAA-producing bacterial endophytes were mainly found in legumes grown in the Herdade da Mitra site. Besides the role of IAA in seed germination and regulation of several developmental and physiological processes in plants [79], it also helps the host plant and microbe to adapt to diverse stress conditions [80,81]. Due to the characteristics of the Herdade da Mitra site [24], it is reasonable to speculate that legumes preferentially associate with strains that possess abilities to help them to cope with the stressful conditions imposed in this site. Similarly, an isolate’s ability to produce siderophores and/or solubilize phosphate may be preferred features for the association of bacteria with plants in such soils. Contrary to what occurs in agricultural fields, in non-cultivated areas there is no input of fertilizers, irrigation or pesticides which makes plants more vulnerable to nutrient deficiencies, water scarcity, and infection. In this sense, the association between native legumes and bacteria able to improve the access and uptake of nutrients would be extremely advantageous for those plants [82,83]. The Herdade da Mitra site possesses manganese toxicity associated with the acid soils [19,84] where there is less availability of stable phosphorous to plants [85] which may explain the presence of the best phosphate-solubilizing endophytes within legumes grown in this site. Nevertheless, further studies to prove the real impact of these plant growth-promoting features on plant growth are needed in order to better understand their importance in legume-bacterial endophytes associations.

Around 10% of the bacterial endophytes tested showed inhibition of *Fusarium oxysporum* sp. growth, with all of them being isolated from one of three native legume species, namely *Ornithopus compressus, Medicago polymorpha,* or *Lupinus angustifolius*, regardless of the legume’s origin. *Fusarium* spp. are ubiquitous soil inhabitants and act as plant pathogens to different legume species, such as *Medicago* spp., *L. angustifolius* and *Cicer arietinum* [86,87,88]. It has been suggested that plants that are exposed to pathogen or insect attack are often able to recruit protective bacteria and, as a result, enhance microbial activity to suppress pathogens in the rhizosphere [89]. Thus, it may be speculated that these legume species associate with the identified bacterial endophytes as a protective measure. Also, only a few ACC deaminase-expressing bacterial endophytes were identified herein. As seen in other studies, the ACC deaminase-expressing bacteria were those that promoted increases in canola root length, reinforcing the importance of this plant growth-promoting trait in plant-bacterial interactions.

As observed in previous studies [24,90], the results observed here suggest that some plant growth-promoting traits may be species-specific whereas others may be strain-related or possibly linked to their endophytic lifestyle. Moreover, the isolates’ very high tolerance to salinity is consistent with what has been reported in other studies [9,24] strengthening the possibility that this may be a common feature related to the endophytic lifestyle of these bacteria. In addition, the isolates mostly were more tolerant to high manganese concentrations than to aluminium. This might be related to the characteristics of the soils in which the plants used as a source of these strains were originally grown and where excessive levels of manganese but not of aluminum are present. In fact, so-called origin soil has previously been suggested as a powerful selective factor in the ecology of endophytic bacterial communities [90,91,92] and is consistent with the general assumption that most endophytes originate from soil [74].

The potential of non-rhizobial endophytic bacteria to impact on the symbiotic performance of two chickpea mesorhizobial strains and, chickpea tolerance to salinity and manganese toxicity conditions were also evaluated in this study. The results show that co-inoculation of chickpea plants with specific endophytic bacteria along with the nitrogen-fixing symbionts can improve the total biomass of chickpea plants, in particular when these plants are grown under saline conditions. However, a negative effect on plants inoculated with a specific endophytic bacterium and UPM-Ca36^T^ symbiont were also obtained under manganese conditions. The potential of endophytic bacteria to improve plant growth and to induce stress tolerance have been reported previously [93,94,95,96,97].

Moreover, our results are in agreement with earlier studies that have also shown that the use of consortia containing rhizobia and non-rhizobial endophytic bacteria benefits both nodulation and nitrogen fixation efficiency [94,96,98,99,100,101]. Each of the four selected endophytic bacteria promoted chickpea biomass in at least one of the conditions tested with one of the mesorhizobial strains. This increase of chickpea biomass is positively related to the total nodule biomass (Figure S2), suggesting that augmentation of nitrogen-fixation occurs by boosting of the mesorhizobial symbiotic performance. However, the augmentation of total nodule dry weight resulted either from an increase of the number of nodules or from the formation of larger nodules. This improved behavior is likely the result of the beneficial functions provided by the endophytic isolates to the plant itself in particular to the plant hormonal homeostasis. Plant hormones play an essential role in plant-microorganism interactions [102] including in the regulation of the nitrogen-fixing symbiotic interactions and the root nodule organogenesis [103]. For instance, auxins and cytokinins are positively involved in the root nodule organogenesis and development while hormones like ethylene, jasmonic acid, abscisic acid and salicylic acid are local inhibitory regulation systems of nodulation i.e., in the control of the number of nodules [103]. Therefore, the IAA synthesis or the reduction of ethylene levels in the chickpea roots, in particular under stressful conditions, may have facilitated the rhizobium-legume interaction under those conditions. In this sense, it is possible that some of the strains, namely *Pseudomonas* sp. D4 and Q1, that displayed ACC deaminase activity under free-living conditions, facilitate nodulation by compatible rhizobia through modulation of ethylene in legume tissues, particularly under stress conditions. In this regard, it has previously been demonstrated that the expression of exogenous ACC deaminase genes under free-living conditions increases the symbiotic performance of many rhizobial strains [104,105] particularly when legumes are grown under stressful conditions [28,49,106] revealing the importance of ACC deaminase in rhizobium-legume symbiosis [107,108]. On the other hand, an augmentation of nodule biomass may be a result of a local accumulation of auxins and cytokinins that stimulate nodule organogenesis and development. It may be possible that the presence of endophytic bacteria has contributed to a balance of auxins and cytokinin that promotes greater development of the root nodules thus leading to a larger number of nitrogen-fixing bacteroids within the nodules. This increase would thus be sufficient to optimize plant nutrition and prevent excessive energy drains on the host plants by forming new nodules. This hypothesis is consistent with the observation that higher nodule dry weight is positively related with chickpea biomass, independent of the number of nodules formed (Figure S2). Recently, it was demonstrated that the endophytic bacterium *Methylobacterium oryzae* mitigates the impact of limited water availability in lentils by increasing plant cytokinin levels [109], while *Sphingomonas* sp. and *Serratia marcescens* endophytes promoted soybean growth through regulation of endogenous hormone content in plants [110]. Although additional studies are needed to better understand how plant-endophyte interactions modulate nodule organogenesis and development, it is possible to speculate that phytohormone balances in plants changed with the presence of bacterial endophytes.

In this study, it was observed that the performance of each individual endophytic bacterium was not identical for the mesorhizobial strains used. These findings are consistent with the notion that the combination of various organisms does not always result in a benefit to the legume [111]. Our previous study showed that isolates assigned to the *M. tianshanense/M. mediterraneum/M. temperatum* cluster, which includes the UPM-Ca36^T^ symbiont, synthesize low amounts of IAA whereas *M. ciceri/M. loti* isolates produced high levels of IAA [90]. In addition, it was also shown that cytokinin production is not associated with any particular mesorhizobial species group, with the UPM-Ca36^T^ symbiont being one of the strains able to synthesize cytokinins. Differences between *Mesorhizobium* strains in these plant growth-promoting features along with others may have contributed to the different plant growth promotion outcomes when co-inoculated with the same endophytic bacterium. Notably, no association with bacterial endophyte origin and the synergistic effects under salinity or Mn toxicity were found. This result may be due to the fact that all strains used were simultaneously tolerant to high concentrations of NaCl and Mn independent of their origin site. Altogether, the differences in the synergistic effects observed here evidence the complexity of tripartite interactions and the importance of a better understanding of these interactions in conditions that better approximates what exists in “true nature” for the development of new and reliable bacterial inoculants for agricultural purposes.

## 5. Conclusions

This study showed that Mediterranean native legume species harbor highly diverse endophytic communities in their root tissues, covering four phyla: Proteobacteria, Firmicutes, Bacteroidetes, and Actinobacteria. Most of these bacteria (83%) possess at least one plant growth-promoting feature and thus might assist the growth and survival of those legume species under Mediterranean conditions. The data also show that the use of consortia containing rhizobia and specific non-rhizobial endophytic bacteria promotes chickpea growth mainly by boosting the nodulation and nitrogen fixation efficiency of the mesorhizobial strains. Therefore, these endophytes may be potential candidates to improve the productivity of legumes worldwide, in particular the ones of agronomic importance [112], that grow in ecological niches with environmental constraints. Nevertheless, differences in the synergistic effects reinforce the notion that without a better understanding of the role of endophytic bacteria, alone and in consortia, their use in sustainable agricultural practices will be poorly informed thereby limiting the development of new and reliable bacterial inoculants for agricultural purposes.

## Figures and Tables

**Table 1:** List of legume species collected in each local and the number of isolates obtained from each legume species in this study.

Origin	Plant Species	Number of Isolates
Herdade da Mitra	*Trifolium* sp.	4
	*Lupinus luteus* L.	3
	*Ornithopus compressus* L.	26
	*Scorpiurus muricatus* L.	4
	*Trifolium subterraneum* L.	5
	*Medicago polymorpha* L.	14
	*Scorpiurus sulcatus* L.	5
	*Trifolium tomentosum* L.	2
	*Ornithopus pinnatus* (Mill.) Druce	3
	*Vicia sativa* L.	8
Alcácer do Sal	*Ornithopus compressus* L.	2
	*Lupinus luteus* L.	4
	*Ornithopus sativus* Brot.	6
	*Lupinus angustifolius* L.	6
	*Medicago polymorpha* L.	22
	*Vicia sativa* L.	8
	Total	122

**Table 2:** Some of the in vitro functional traits of the isolated bacterial endophytes. All of the isolates were scored as either having activity (1) or not (0) having activity, therefore the numbers indicate the number of isolates from each genus that expresses the indicated trait. Green shading indicates that <25% of the isolates exhibited medium or high levels of that trait, yellow indicates 25−50% of the isolates exhibited medium or high levels of that trait, orange indicates 50−75% of the isolates exhibited medium or high levels of that trait, and red indicates 75−100% of the isolates exhibited medium or high levels of that trait.

Bacterial Genera	Number of Isolates	IAA Production	Cellulase Production	Siderophore Production	Phosphate Solubilization
*Microbacterium*	1	0	1	0	0
*Chryseobacterium*	4	4	1	2	0
*Bacillus*	26	15	15	1	2
*Paenibacillus*	2	2	2	1	0
*Staphylococcus*	2	2	0	2	1
*Pantoea*	5	5	1	0	1
*Erwinia*	2	2	0	0	1
*Achromobacter*	1	0	0	0	0
*Lelliottia*	1	1	0	0	1
*Enterobacter*	1	0	0	0	0
*Acinetobacter*	1	0	0	0	0
*Janthinobacterium*	1	0	0	1	0
*Pseudomonas*	49	16	30	28	19
*Stenotrophomonas*	8	5	1	3	0
*Serratia*	15	11	0	1	1
*Rahnella*	3	3	1	0	1
Total	122	66	52	39	27

**Table 3:** Tolerance of endophytic bacteria to Al, Mn, and NaCl. * Data from [24]

Origin Site	Origin Legume Species	Endophytic Bacterial Isolate	Maximal Concentration Tolerated		
			NaCl (mM)	Mn (mM)	Al (mM)
Herdade da Mitra	*Trifolium* sp.	*Bacillus* sp. A1	1275	0.1	0.1
	*Lupinus luteus*	*Bacillus* sp. B2	850	2.5	2.5
	*Lupinus luteus*	*Pantoea* sp. B3	1275	1	2.5
	*Scorpiurus muricatus*	*Pseudomonas* sp. D4	850	2.5	1
	*Trifolium subterraneum*	*Bacillus* sp. F5	850	2.5	2.5
	*Medicago polymorpha*	*Serratia* sp. G1	85	0.5	2.5
	*Medicago polymorpha*	*Paenibacillus* sp. G2	850	1	2.5
	*Trifolium tomentoseum*	*Pseudomonas* sp. I2	1275	2.5	0.1
	*Ornithopus compressus*	*Pseudomonas* sp. L13	850	2.5	2.5
	*Vicia sativa*	*Bacillus* sp. M4	850	2.5	2.5
Alcácer do Sal	*Lupinus angustifolius*	*Pseudomonas* sp. Q1	850	2.5	0.1
	*Lupinus angustifolius*	*Pseudomonas* sp. Q2	85	2.5	1
	*Lupinus angustifolius*	*Serratia sp*. Q5	850	2.5	1
	*Lupinus angustifolius*	*Rahnella* sp. Q6	1700	2.5	2.5
	*Medicago polymorpha*	*Erwinia* sp. T5	1700	2.5	0.05
	Cicer arietinum *	*Kosakonia* sp. MH5 *	850 *	1 *	2.5

**Table 4:** The effect of endophytic bacteria in combination with *M. ciceri* LMS-1 or *M. mediterraneum* UPM-Ca36^T^ symbionts on chickpea biomass, nodule dry weight (NDW) and number of nodules (NN), under control, salinity and manganese conditions. # indicates statistically significant differences between positive controls (*p* < 0.05); ** indicates statistically significant differences between co-inoculated treatments and single inoculated treatments (*p* < 0.05).

Condition	Treatment	Biomass	NDW	NN
Control	Positive Control	2.561 ± 0.102	0	0
	LMS-1	1.370 ± 0.040	0.143 ± 0.004	79.8 ± 7.4
	LMS-1 + D4	1.378 ± 0.036	0.134 ± 0.003	58.2 ± 3.0 **
	LMS-1+ Q1	1.549 ± 0.036	0.164 ± 0.022	69.3 ± 5.5
	LMS-1 + MH5	1.552 ± 0.097	0.173 ± 0.019	70.5 ± 4.4
	LMS-1 + L13	1.575 ± 0.075 **	0.167 ± 0.018	58.3 ± 1.0 **
	Ca36	1.295 ± 0.048	0.195 ± 0.015	84.2 ± 4.7
	Ca36 + D4	1.298 ± 0.029	0.185 ± 0.020	60.8 ± 6.1 **
	Ca36 + Q1	1.204 ± 0.081	0.198 ± 0.016	69.5 ± 8.8
	Ca36 + MH5	1.269 ± 0.057	0.198 ± 0.010	72.3 ± 14.1
	Ca36 + L13	1.363 ± 0.037	0.196 ± 0.011	78 ± 8.3
Salt	Positive Control	1.605 ± 0.179 #	0	0
	LMS-1	0.923 ± 0.019	0.083 ± 0.006	54.6 ± 9.1
	LMS-1 + D4	0.925 ± 0.075	0.076 ± 0.012	78.3 ± 3.1
	LMS-1 + Q1	1.036 ± 0.037 **	0.088 ± 0.007	70.8 ± 8.4
	LMS-1 + MH5	1.259 ± 0.102 **	0.104 ± 0.009	98.5 ± 10.2 **
	LMS-1 + L13	1.151 ± 0.080 **	0.093 ± 0.012	89.7 ± 4.8 **
	Ca36	0.787 ± 0.043	0.083 ± 0.004	68.0 ± 12.4
	Ca36 + D4	1.087 ± 0.128 **	0.128 ± 0.014 **	104.5 ± 20.7
	Ca36 + Q1	0.997 ± 0.045 **	0.199 ± 0.006 **	100.2 ±17.3
	Ca36 + MH5	0.768 ± 0.048	0.093 ± 0.004	76.0 ± 18.3
	Ca36 + L13	0.989 ± 0.047 **	0.110 ± 0.004 **	72.6 ± 10.4
Mn	Positive Control	2.031 ± 0.143 #	0	0
	LMS-1	0.575 ± 0.044	0.043 ± 0.005	52.0 ± 9.8
	LMS-1 + D4	0.685 ± 0.080	0.048 ± 0.008	53.7 ± 6.3
	LMS-1 + Q1	0.695 ± 0.067	0.042 ± 0.010	52.5 ± 3.4
	LMS-1 + MH5	0.500± 0.044	0.036 ± 0.005	59.0 ± 9.4
	LMS-1 + L13	0.572 ± 0.064	0.041 ± 0.008	54.0 ± 15.3
	Ca36	0.688 ± 0.043	0.089 ± 0.003	50 ± 2.6
	Ca36 + D4	0.528 ± 0.041 **	0.060 ± 0.009 **	31.8 ± 3.2 **
	Ca36 + Q1	0.972 ± 0.035 **	0.120 ± 0.005 **	47.0 ± 4.9
	Ca36 + MH5	0.636 ± 0.013	0.100 ± 0.004	61.0 ± 11.9
	Ca36 + L13	0.807 ± 0.082	0.112 ± 0.009 **	60.2 ± 5.3

## Supplementary Materials

The following are available online at https://www.mdpi.com/2076-2607/7/10/392/s1, Table S1. Results obtained for cellulase activity, IAA synthesis, siderophore production, phosphate solubilization and antifungal activity.; Cellulase activity classes: 0—no activity; 1—(>0 and ≤0.5 cm); 2—(>0.5 and ≤1 cm) and 3—(>1 cm); n.d.—not determined. Accession number for partial 16S rRNA gene sequence. Figures S1 and S2.
