# Supplementary material for: Mediterranean Native Leguminous Plants: A Reservoir of Endophytic Bacteria with Potential to Enhance Chickpea Growth under Stress Conditions
Source: Microorganisms. 2019 Sep 25;7(10):392. doi: 10.3390/microorganisms7100392 (PMC6843138; doi:10.3390/microorganisms7100392)
Supplement: Supplementary file 1 [file microorganisms-07-00392-s001.zip › Table S1.pdf]

Table S1. Results obtained for cellulase activity, IAA synthesis, siderophore production, phosphate solubilization and antifungal activity.; Cellulase activity classes: 0 - no activity; 1 - (> 0 and ≤ 0.5 cm); 2 - (> 0.5 and ≤ 1 cm) and 3 - (> 1 cm); n.d. - not determined. Accession number for partial 16S rRNA gene sequence.

**Origin**	**Legume species**	**Isolate**	**IAA production [µg/ml]**	**Cellulase activity class**	**ACC deaminase [µmol α-ketobutyrate/mg protein/h]**	**Antifungal activity**	**Siderophores production index**	**Phosphate solubilization index**	**Accession number**
Alcácer do Sal	*Lupinus angustifolius* L.	**Q1**	0	2	8,75	yes	2,681	1,222	MK007434
		**Q2**	0	1	2,50	yes	3,000	1	MK007435
		**Q3**	0	0	0	-	1	1	MK007436
		**Q4**	1,4	n.d.	0	-	1	1	MK007437
		**Q5**	1,1	0	0	yes	1	1	MK007438
		**Q6**	41,6	2	0	yes	1	1,238	MK007439
	Lupinus luteus L.	**O1**	4,4	0	0	-	1	1	MK007424
		**O2**	0,5	1	0	-	1	2,111	MK007425
		**O3**	3	1	0	-	1	1	MK007426
		**O4**	0	1	0	-	1	1,867	MK007427
	Medicago polymorpha L.	**R1**	22,7	n.d.	2,58	-	3,625	1	MK007440
		**R2**	0	1	0	-	1,287	1	MK007441
		**R3**	0	2	0	yes	1	1	MK007442
		**R4**	5,3	0	0	-	1	1	MK007443
		**R5**	0	1	0	-	1,203	1,300	MK007444
		**R6**	0	2	0	-	1	1	MK007445
		**R7**	0	1	0	-	1,258	1,400	MK007446
		**R8**	0	0	0	yes	1	1	MK007447
		**T1**	0,2	0	0	-	1	1	MK007456

	**T10**	0	0	0	-	1	1	MK007465
	**T11**	0	0	2,07	-	1	1,292	MK007466
	**T12**	0	0	0	-	1	1	MK007467
	**T13**	1,5	0	0	-	1	1	MK007468
	**T14**	0,7	n.d.	0	-	1	1	MK007469
	**T2**	0	0	0	-	1,285	1,333	MK007457
	**T3**	1,4	2	0	-	1,239	1,206	MK007458
	**T4**	0	0	0	-	1	1	MK007459
	**T5**	54	0	0	yes	1	1,587	MK007460
	**T6**	0	1	0	-	1,267	1,237	MK007461
	**T7**	0,2	1	0	-	1	1,237	MK007462
	**T8**	0	0	0	-	1	1	MK007463
	**T9**	1,8	1	0	-	1	1	MK007464
Ornithopus compressus L.	**N1**	0,1	0	0	-	1	1	MK007422
	**N2**	0	0	0	-	1	1	MK007423
Ornithopus sativus Brot.	**P1**	22	n.d.	0	-	4,037	1	MK007428
	**P2**	0	1	0	-	1	1,550	MK007429
	**P3**	0	1	0	-	1,276	1	MK007430
	**P4**	0	3	0	-	1	1	MK007431
	**P5**	5,1	0	0	-	1	1	MK007432
	**P6**	n.d.	n.d.	0	-	n.d.	n.d.	MK007433
Vicia sativa L.	**S1**	0	1	0	-	1	1	MK007448
	**S2**	0	0	0	-	2,540	1	MK007449
	**S3**	0	1	0	-	1	1	MK007450
	**S4**	0,4	0	0	-	1,829	1	MK007451
	**S5**	2,3	0	0	-	1	1,322	MK007452
	**S6**	0,8	n.d.	0	-	1	1	MK007453

		**S7**	0	0	0	-	1	1,400	MK007454
		**S8**	0	0	0	-	2,167	1	MK007455
Herdade da Mitra	*Lupinus luteus* L.	**B1**	0	2	0	-	1	1	MK007352
		**B2**	0	3	0	-	1	1	MK007353
		**B3**	59,1	0	0	-	1	2,167	MK007354
	Medicago polymorpha L.	**G1**	7,2	0	0	yes	1	1	MK007379
		**G2**	11,7	3	0	yes	4,506	1	MK007380
		**G4**	7,5	3	0	-	1	1	MK007381
		**G5**	8,1	0	0	-	1	1,583	MK007382
		**G6**	3,4	0	0	-	1	1	MK007383
		**J1**	n.d.	n.d.	0	-	1,333	1	MK007391
		**J2**	11,2	3	0	-	1	1	MK007392
		**J3**	1,8	1	0	-	1	1	MK007393
		**J4**	2,5	2	0	-	1	1,917	MK007394
		**J5**	1,8	0	0	-	1	1,250	MK007395
		**J6**	97,1	2	0	-	1	1	MK007396
		**J7**	1,7	1	0	-	1	1,667	MK007397
		**J8**	2,3	2	0	-	1	1	MK007398
		**J9**	0,8	2	0	-	1	1	MK007399
	Ornithopus compressus L.	**C1**	12,3	0	0	-	1	1	MK007355
		**C2**	6,2	0	0	-	1	1	MK007356
		**C3**	0	3	0	-	1	1	MK007357
		**C4**	0	0	0	-	1	2,000	MK007358
		**C5**	0	0	0	-	1,159	1,917	MK007359
		**E1**	0	1	0,70	-	1	1,667	MK007364
		**E2**	0,1	0	0	-	1	1	MK007365
		**E3**	48,0	1	0	yes	1	1,889	MK007366
		**E4**	0	1	0	-	1,277	1	MK007367

	**E5**	67,3	0	0	-	1	1	MK007368
	**E6**	0	2	0	-	1,108	1	MK007369
	**E6b**	10,7	0	0	-	1	1	MK007370
	**E7**	0	0	0	yes	1,450	1	MK007371
	**E8**	0,5	0	0	-	1	1	MK007372
	**E9**	0	n.d.	0	-	2,079	1	MK007373
	**L1**	55,3	0	0	-	1	1	MK007403
	**L10**	0	3	0	-	1,203	1	MK007410
	**L11**	0,3	0	0	-	1,075	1	MK007411
	**L12**	14,9	1	0	-	1	1	MK007412
	**L13**	0	0	0	yes	2,232	1	MK007413
	**L3**	0,3	0	0	-	1	1	MK007404
	**L4**	0	1	0	-	1,443	1	MK007405
	**L5**	59,0	0	0	yes	1,305	1	MK007406
	**L7**	0	2	0	-	1,344	1,917	MK007407
	**L8**	0	3	0	-	1	1	MK007408
	**L9**	0	2	0	-	1,421	1	MK007409
Ornithopus pinnatus (Mill.) Druce	**K1**	0,7	1	0	-	1,401	1	MK007400
	**K2**	36,3	0	0	-	1	1	MK007401
	**K3**	47	0	0	-	1	1	MK007402
Scorpiurus muricatus L.	**D1**	0	0	0	-	1	1	MK007360
	**D2**	0	0	0	-	1	1	MK007361
	**D3**	27,5	0	0	-	1	1	MK007362
	**D4**	0	1	2,01	-	2,778	1	MK007363
Scorpiurus sulcatus L.	**H1**	14,7	0	0	-	1,917	1	MK007384
	**H2**	0,6	0	0	-	2,833	1	MK007385
	**H3**	0,8	1	0	-	1,944	1	MK007386
	**H4**	8,7	0	0	-	1	1	MK007387

	**H5**	0	0	0	-	1	1	MK007388
Trifolium sp.	**A1**	9,3	2	0	-	1	1	MK007348
	**A2**	0	0	1,23	-	1,341	1	MK007349
	**A3**	36,7	0	0	-	1,247	1	MK007350
	**A4**	0	0	0	-	1	1	MK007351
Trifolium subterraneum L.	**F1**	0	0	0	-	1,750	1	MK007374
	**F2**	1	0	0	-	1	1	MK007375
	**F3**	0,7	0	0	-	1	1	MK007376
	**F4**	0	0	0	-	1,268	1,417	MK007377
	**F5**	8,4	3	0	-	1	1	MK007378
Trifolium tomentosum L.	**I1**	37,4	2	0	-	1	1	MK007389
	**I2**	0	1	0	-	1,500	1	MK007390
Vicia sativa L.	**M1**	4,4	2	0	-	1	1	MK007414
	**M2**	0,2	0	0	-	1	1	MK007415
	**M3**	14,7	0	0	-	1,132	2,333	MK007416
	**M4**	4,1	3	0	-	1	1	MK007417
	**M5**	0	3	0	-	1	1	MK007418
	**M6**	0	0	0	-	1	1	MK007419
	**M7**	4,1	0	0	-	1	1	MK007420
	**M8**	n.d.	0	1,22	-	1	1	MK007421
