# Supplementary figures and images for: Mediterranean Native Leguminous Plants: A Reservoir of Endophytic Bacteria with Potential to Enhance Chickpea Growth under Stress Conditions
Source: Microorganisms. 2019 Sep 25;7(10):392. doi: 10.3390/microorganisms7100392 (PMC6843138; doi:10.3390/microorganisms7100392)

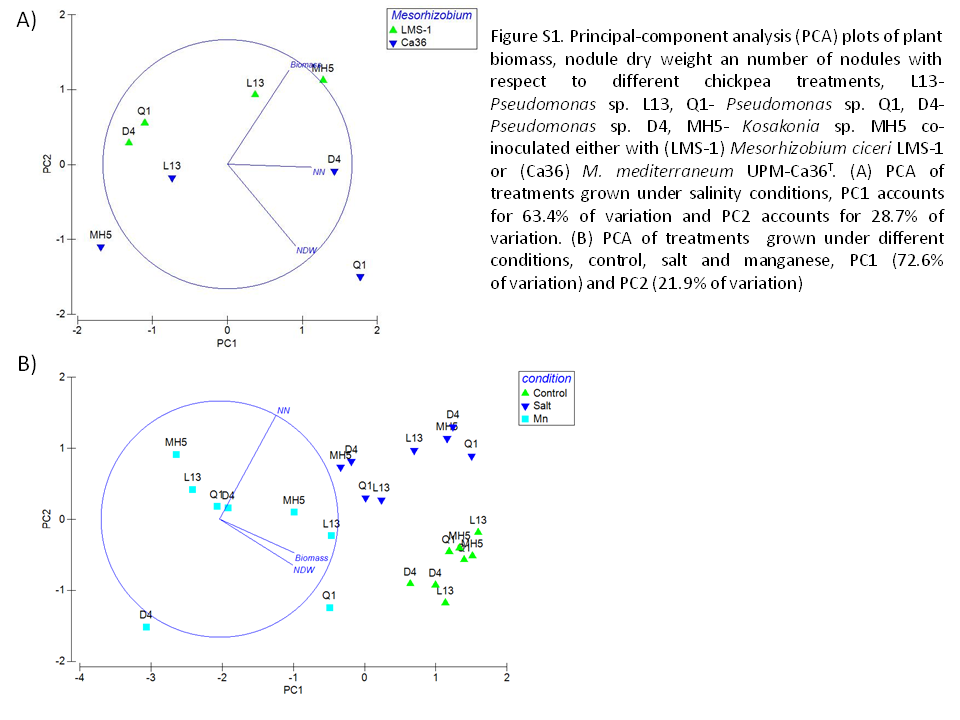

Supplement: Supplementary file 1 [file microorganisms-07-00392-s001.zip › Figure S1.tif]

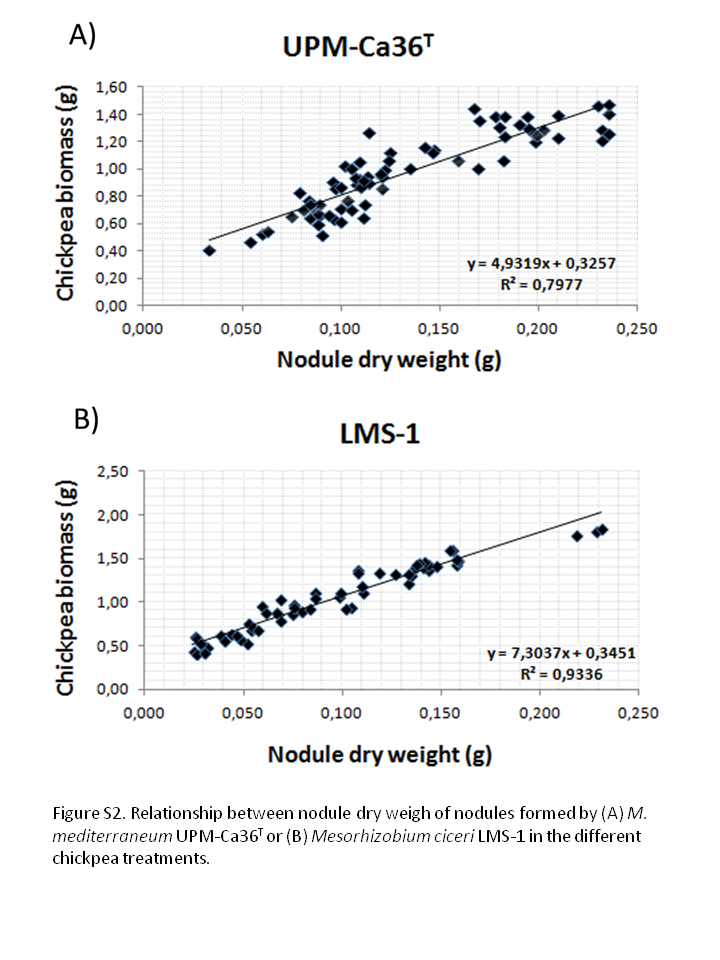

Supplement: Supplementary file 1 [file microorganisms-07-00392-s001.zip › Figure S2.tif]
